# A comprehensive signature based on endoplasmic reticulum stress-related genes in predicting prognosis and immunotherapy response in melanoma

**DOI:** 10.1038/s41598-023-35031-9

**Published:** 2023-05-22

**Authors:** Longqing Liu, Dilang Yao, Zhiwei Chen, Shidong Duan

**Affiliations:** Department of Otolaryngology Head and Neck Surgery, Enshi Prefecture Ethnic Hospital, 178 Hangkong Avenue, Enshi, Hubei Province China

**Keywords:** Cell death and immune response, Oncology

## Abstract

Melanoma is considered as one of the most invasion types of skin cancer with high mortality rates. Although combination of immune checkpoint therapy with local surgical excision provide a novel promising therapeutic strategies, the overall prognosis of melanoma patients remains unsatisfactory. Endoplasmic reticulum (ER) stress, a process of protein misfolding and undue accumulation, has been proven to play an indispensable regulatory role in tumor progression and tumor immunity. However, whether the signature based ER genes has predictive value for the prognosis and immunotherapy of melanoma has not been systematically manifested. In this study, the LASSO regression and multivariate Cox regression were applied to construct a novel signature for predicting melanoma prognosis both in the training and testing set. Intriguingly, we found that patients endowed with high- and low-risk scores displayed differences in clinicopathologic classification, immune cell infiltration level, tumor microenvironment, and immune checkpoint treatment response. Subsequently, based on molecular biology experiments, we validated that silencing the expression of RAC1, an ERG composed of the risk signature, could restrain the proliferation and migration, promote apoptosis, as well as increase the expression of PD-1/PD-L1 and CTLA4 in melanoma cells. Taken together, the risk signature was regarded as promising predictors for melanoma prognosis and might provide prospective strategies to ameliorate patients’ response to immunotherapy.

## Introduction

Melanoma is considered as one of the most invasive types of cancer with the highest mortality rate although it accounts for less than 5% of all skin cancers^1^. The highly metastatic characteristics confer a poor outcome of melanoma patients, where the survival rate of patients at stage IV decreases from 92 to 19% in 5 years^2^. Therefore, given the high malignancy and ﻿tumor heterogeneity, there is an urgent need to seek an effective predictor in melanoma prognosis and potential targets for melanoma treatment.

The endoplasmic reticulum (ER), the largest organelle in eukaryotic cells, is composed of a highly dynamic network of tubules and branches and participates in the biosynthesis, processing and trafficking of proteins^3^. Once the protein folding machinery in the ER is perturbed by various stress signals, including intracellular and extracellular factors, the unfolded/misfolded proteins will excessively accumulate in the lumen of ER, which is known as ER stress^4^.

Notably, the augment of ER stress could influence the proliferation, invasion, ﻿angiogenesis and survival of tumor cells^5^. In melanoma cells, the genetic mutation and hypermetabolic environment led to increasing demands for protein synthesis, causing ER stress^6^. The ER stress and UPR maintenance in melanoma cells affected cellular reprogramming, thus dominating cancer initiation, influencing progression, and inducing resistance-related cellular phenotypes. Importantly, the intensity and duration of ER stress might cause cell fate towards pro-survival or pro-apoptosis. Oncogenic BRAF activated MEK/ERK signaling pathway, increased the protein folding load in the ER and induced ER stress, thus promoting melanoma malignant progression^7^. Meanwhile, the un-restored ER homeostasis could induce apoptosis while not inducing cytoprotective adaptation^4^. The induction of excessive ER stress to stimulate adaptive UPR signaling might serve as a promising strategy to diminish melanoma cells^8^. Furthermore, recent studies unravel that ER stress is also associated with the prognosis of various cancers via pro-tumorigenic or anti-tumorigenic effects, including glioma^9^, pulmonary adenocarcinoma^10^, and hepatocellular carcinoma^11^. Moreover, Fan et al.^12^ verified that the low expression of stress-associated endoplasmic reticulum protein 1 (SERP1), a gene induced by ER stress, was related to poor prognosis and immune infiltration in skin cutaneous melanoma. In addition, Liu et al.^13^ established a prognostic model based on ER stress-related genes (ERGs) to successfully predict the prognosis of hepatocellular carcinoma patients. However, there have reported no studies to construct a predictive signature based on ERGs for melanoma prognosis.

Hence, in our study, we firstly identified 787 ERGs from The Cancer Genome Atlas (TCGA) project. Then, by using the Cox regression analysis, nine ERGs were finally confirmed to calculate the risk score and construct a risk signature. The prognostic value and predictive ability of the risk signature in melanoma patients were validated by Kaplan–Meier survival analysis and receiver operating characteristic (ROC) curves. Besides, the univariate and multivariate cox regression analyses and development of a nomogram were used to evaluate the independent capacity of the risk score. The optimal predictive performance of the risk signature was manifested through comparison with other external signatures. Furthermore, we explored the differential immunity and response to immunotherapy and chemotherapy by using the ESTIMATE algorithm, immunophenoscore (IPS) score, SubMAP algorithm and Tumor Immune Dysfunction and Exclusion (TIDE) algorithm. Of note, we validated the role of RAC1, an ERG, in modulating the behavior of melanoma cells and regulating the expression of programmed cell death-1 (PD-1)/programmed death-ligand 1 (PD-L1) and cytotoxic T-lymphocyte-associated antigen 4 (CTLA4) in vitro. Collectively, we tended to clarify the value of the ERGs-based signature in predicting melanoma prognosis and improving the response to immune treatment.

## Materials and methods

### Data acquisition and random grouping

The normalized RNA sequencing data (FPKM format) of ERGs and the corresponding clinical features of 471 melanoma samples, could be downloaded from the TCGA data portal (https://portal.gdc.cancer.gov/). The inclusion of patients were according to criteria as: (1) The patients were diagnosed as melanoma; (2) The case had complete expression profiles and clinical information. The exclusion of patients were according to criteria as: (1) patients without survival information or survival time was less than 30 days; (2) patients without clinical information. Finally, 458 melanoma samples were set as a training set, and 230 melanoma patients were randomly selected as an internal testing set. Table S1 showed the clinical features of enrolled melanoma patients. 210 melanoma samples from the GSE65904 dataset were used as external testing set.

### Construction of the risk signature based on ERGs

Firstly, the univariate Cox regression analysis was used to identify ERGs associated with overall survival (OS) in melanoma patients. Next, the Least Absolute Shrinkage and Selection Operator (LASSO) Cox regression analysis was performed to reduce the number of genes by using the “glmnet” software package. The multivariate Cox regression analysis was stepwise used to identify the final predictors with nonzero coefficients. The following formula was used to calculate the risk score: the risk score = Expression_ERG(1)_ × Coefficient_ERG(1)_ + Expression_ERG(2)_ × Coefficient_ERG(2)_ + . . . + Expression _ERG(n)_ × Coefficient _ERG(n)_. Patients in the training and testing set were divided into high-risk and low-risk groups according to the median cutoff of the risk score. The Kaplan–Meier survival curves were drawn to estimate the survival time of melanoma patients by using the “survival” package in R. The “survivalROC” R package was used to plot the ROC curve to evaluate the sensitivity and specificity of the signature on OS in BC patients at 1-, 2-, and 3-year. The ROC curve was plotted by ﻿using the “survivalROC” R package to calculate the area under the curve (AUC) value, which was used to evaluate the predictive performance of factors in predicting survival at 1-, 2-, and 3-year.

### Validation of the prognostic value of the risk score

Univariate and multivariate Cox regression analyses were performed to identify the independent prognostic factors. The “rms” R package was used to build a nomogram to predict the 1-, 2-, and 3-year survival rate of melanoma patients. The calibration curve was plotted for comparison with the actual observation to estimate the effect and accuracy of the nomogram. The C-index was calculated by ﻿using the “rms” package in R to assess the prognostic prediction performance of the signature.

### The immune analysis

The CIBERSORT package was used to estimate the proportions of 22 immune cell subtypes. The results with P < 0.05 were used for further analysis. The single-sample gene set enrichment analysis (ssGSEA) was used to evaluate the activities of 13 immune-related pathways by using the “GSEAbase” R package. The ESTIMATE algorithm was used to analyze the Immune score, tumor purity, ESTIMATE score, and stromal score for each melanoma patient further to estimate the immune cell and stromal cell abundance.

### The analysis of immunotherapy and chemotherapy differences

IPS, including four different immune-phenotypes (effector cells, immunosuppressive cells, MHC molecules, and immunomodulators), could be used to predict the response of patients to the immune checkpoint inhibitor (ICI) treatment^14^. The IPS, ranging from 0 to 10, was calculated to quantitatively determine the immunogenicity by using machine learning methods. The SubMAP algorithm was used to predict the response to anti-PD1 and anti-CTLA4 immunotherapy in high-risk and low-risk groups. The cancer-immunity cycle, mainly including seven steps, is a critical framework for tumor immunotherapy study. The data of genes from each step could be obtained from Tracking Tumor Immunephenotype (http://biocc.hrbmu.edu.cn/TIP/index.jsp). The ssGSEA algorithm was used to quantify the scores of the seven steps. Then the cancer-immunity scores were compared between high- and low-risk groups. Meanwhile, the TIDE algorithm (http://tide.dfci.harvard.edu/) was used to predict the response to ICI treatment. ﻿Patients who got higher TIDE prediction scores could get higher chances for the genesis of immune escape and benefited less from ICI therapy. The “pRRophetic” and “ggplot2 ” package in R was used to compare the 50% inhibitory concentration (IC50%) values of commonly used chemotherapeutic drugs in patients with high- and low-risk scores. The data about IC50 values could be obtained by using high-throughput sequencing data of melanoma in the TCGA project.

### The in vitro validation of the function of RAC1

#### Cell culture and transfection

The human melanoma cell lines A375 and A875 were purchased from China Infrastructure of Cell Line Resource and routinely cultured in Dulbecco’s modified Eagle’s medium (DMEM) supplemented with 10% FBS at 37 °C in a humidified environment with 5% CO_2_. The siRNAs of RAC1 and the corresponding negative control were purchased from Ribobio (Wuhan, China). The melanoma cells were firstly cultured in the 6-well plate and then transfected with 50 nM siRAC1 or siNC by using Lipofectamine 3000 reagent (Invitrogen, Shanghai, China) based on the corresponding instructions. Three independent experiments were conducted.

#### Cell proliferation assay

The proliferation of cells was measured via the CCK-8 (Dojindo, Kumamoto, Japan) assay. The transfected cells were seeded in the 96-well plate at a density of 2 × 10^3^/well, and the cell viability was detected from 12 to 60 h by directly adding CCK-8 reagent to each well. The optical density (OD) of each well was recorded at a wavelength of 450 nm using a microplate reader (BioTek Instruments, United States). Three independent experiments were conducted.

#### Scratch/wound healing assay

After transfection with siNC or siRAC1 for 24 h, A375 and A875 cells were plated in 6-well plates at a density of 3000 cells per well. When the cells ﻿were cultured to 90–95% confluence, a linear wound across the confluent cell layer was scraped by using a sterile 200 μl pipette head. Cells were washed gently thrice with PBS to remove floated cells and debris. The wounds of scratch were observed under the ﻿BX41 light microscope (Olympus, Japan) at 0 h and 24 h after scratches. Relative migration distance = gap distance at 0 h−gap distance at 24 h. Three independent experiments were conducted.

#### Cell apoptosis assay

After transfection with siNC or siRAC1 for 24 h, the treated A375 and A875 cells were firstly digested with trypsin without EDTA. Then after washing with PBS thrice, the cells were stained with Annexin V-FITC and propidium iodide (PI) according to corresponding instructions. Apoptotic cells were measured by the flow cytometer (BD Biosciences, NJ). Three independent experiments were conducted.

#### qRT-PCR analysis

Total RNA was extracted from A375 and A875 cells by using the TRIzol reagent kit (Invitrogen/Thermo Fisher Scientific). The concentration of RNA was measured by a K5800 spectrophotometer (Kaiao, Beijing, China). Then, the RNA was reversely transcribed into complementary DNA (cDNA) using the PrimeScript RT Master Mix (Takara Bio, inc) and then subjected to qPCR using the SYBR Premix Ex Taq™ (Takara). The expression level of GAPDH was used as an internal standard control. All the gene expression levels were collected and quantified by using the 2^−△△Ct^ method. The primer sequences were provided in Table S2. Three independent experiments were conducted.

### Statistics analysis

All statistical analysis was performed by using R version 4.0.5 and GraphPad Prism (version 8.0). The independent t test was used to compare the continuous variables between two groups. The χ^2^-test was used to compare the differences in proportions. The Wilcoxon test was conducted to compare the TIDE score between groups. Pearson correlation analysis was used to estimate the correlation between two variables. All statistical tests were two-sided, and the values of P < 0.05 were considered to be significant.

## Results

### Construction of a risk model based on ERGs in melanoma

**Figure 1:**
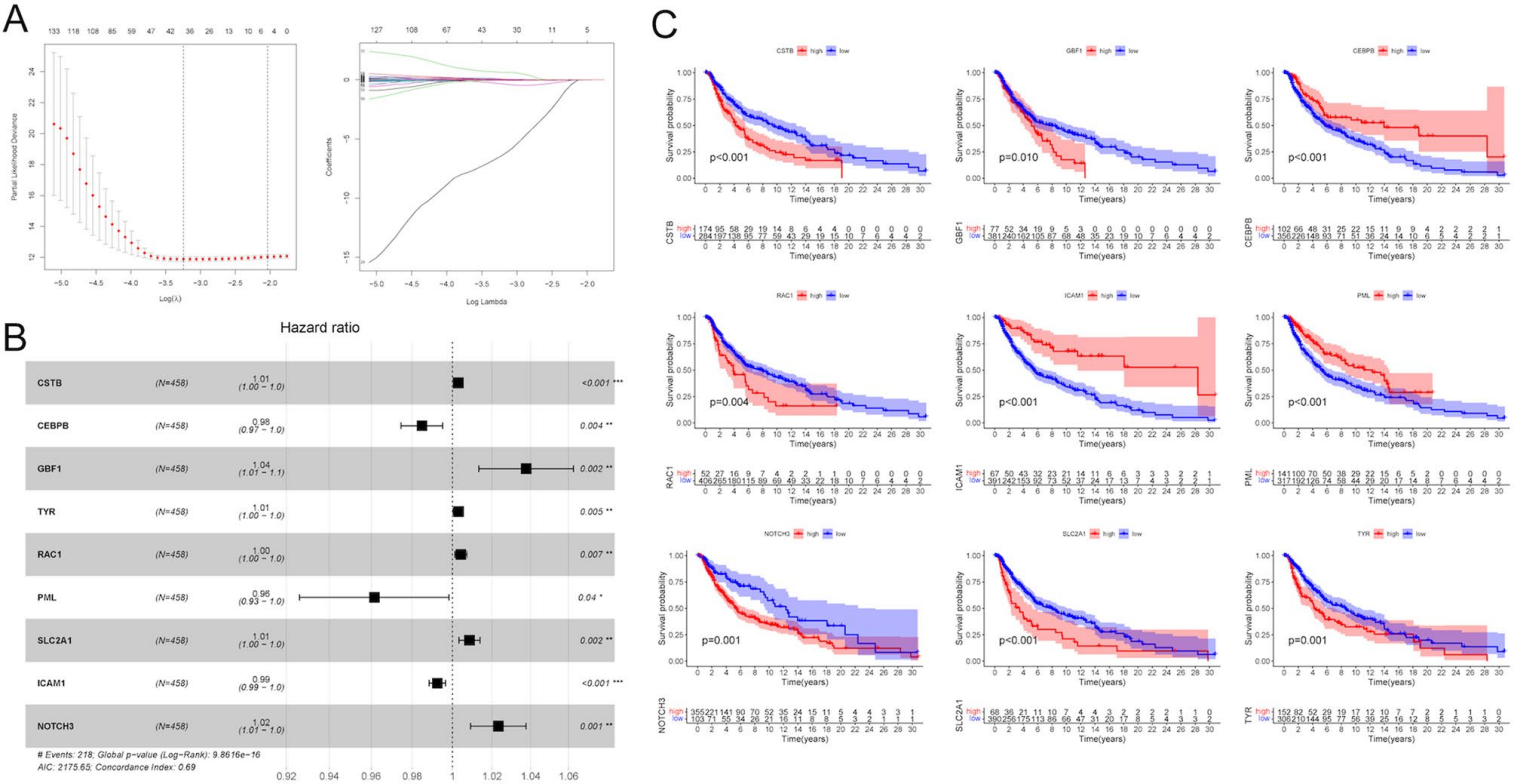
Construction of the risk score based on ERGs. (**A**) LASSO regression analysis of ERGs. (**B**) The forest plot of the HR for the correlation between ERGs and melanoma prognosis. (**C**) The Kaplan–Meier survival curves of melanoma patients with high and low expression of ERGs.

A total of 787 ERGs were identified in 458 melanoma patients from the TCGA project (Table S3). The expression profiles of these 787 ERGs in the training cohort were used to construct a prognostic model. Firstly, the univariate Cox regression was used to identify 249 ERGs that were significantly associated with OS of melanoma patients (P < 0.05) (Table S4). Then, 249 ERGs were thrown to perform LASSO Cox regression analysis, and 40 ERGs were identified (Fig. 1A). Next, the multivariate Cox regression analysis was used to further reduce the number of genes. The forest plot of the multivariate Cox regression analysis showed that nine ERGs were finally utilized to establish the risk model, including CSTB, CEBPB, GBF1, TYR, RAC1, PML, SLC2A1, ICAM1 and NOTCH3 (Fig. 1B). The risk score = CSTB × (0.001805) + CEBPB × (− 0.015339) + GBF1 × (0.037043) + TYR × (0.000855) + RAC1 × (0.004245) + PML × (− 0.039240) + SLC2A1 × (0.008513) + ICAM1 × (− 0.007551) + NOTCH3 × (0.023026). The Kaplan–Meier survival analysis showed that the expression of these nine ERGs was significantly related to the survival time of melanoma patients (P < 0.05) (Fig. 1C).

### Validation of the prognostic value of the risk score in the training and testing sets

**Figure 2:**
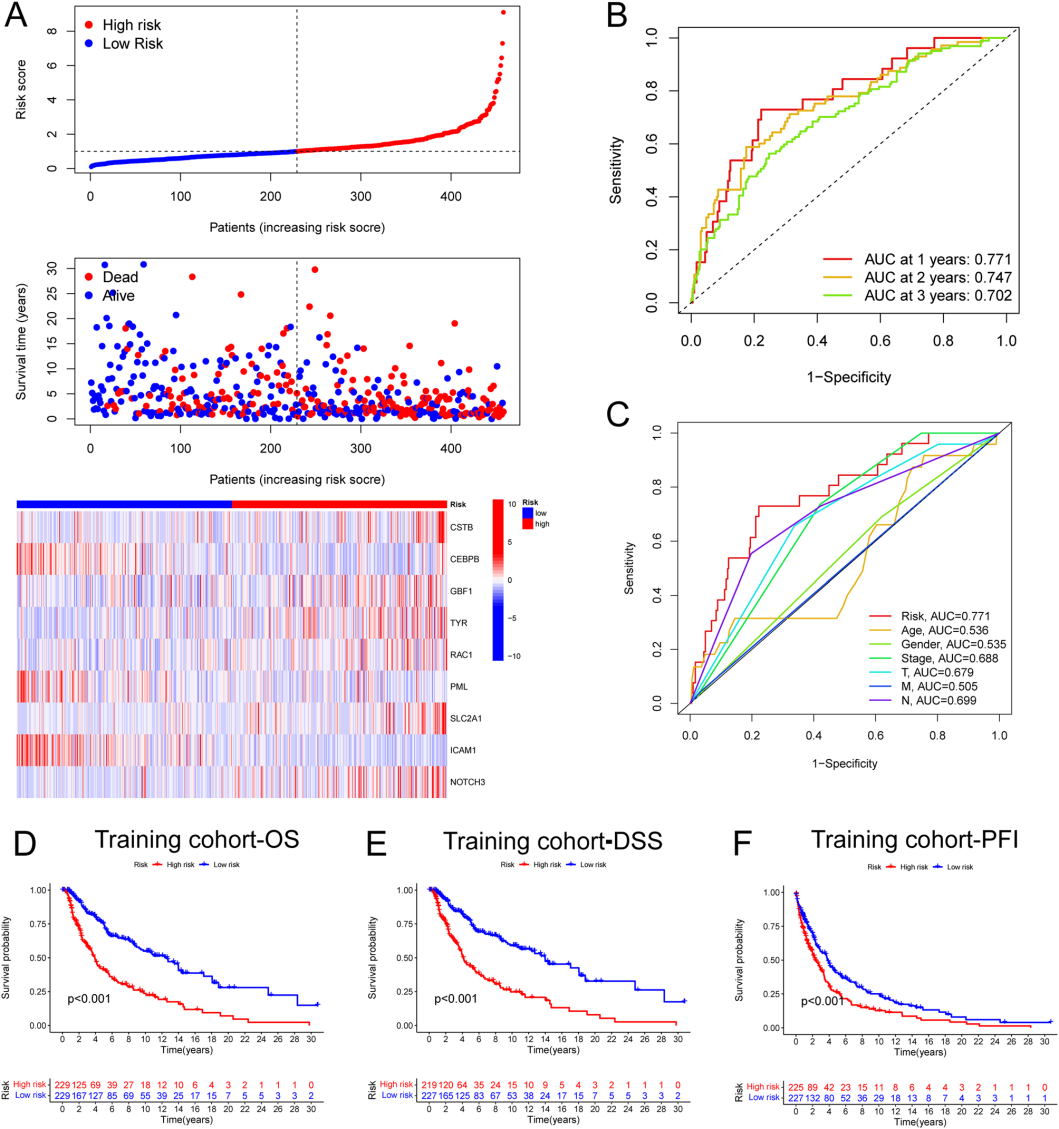
The prognostic value of the risk score in the training set. (**A**) The risk curve and scatterplot based on the risk score and survival status of each melanoma sample. Besides, the heatmap showed the expression levels of ERGs in the high-risk and low-risk groups. (**B**) The AUC for the risk score at 1-, 2- and 3 years according to the ROC curves. (**C**) The AUC for the risk score and other clinical features at 1-year according to the ROC curves. (**D**) The Kaplan–Meier survival analysis showed the OS, the PFI, and the DSS of melanoma patients between high- and low-risk groups. OS, overall survival. PFI, progression-free interval. DSS, disease-specific survival.

**Figure 3:**
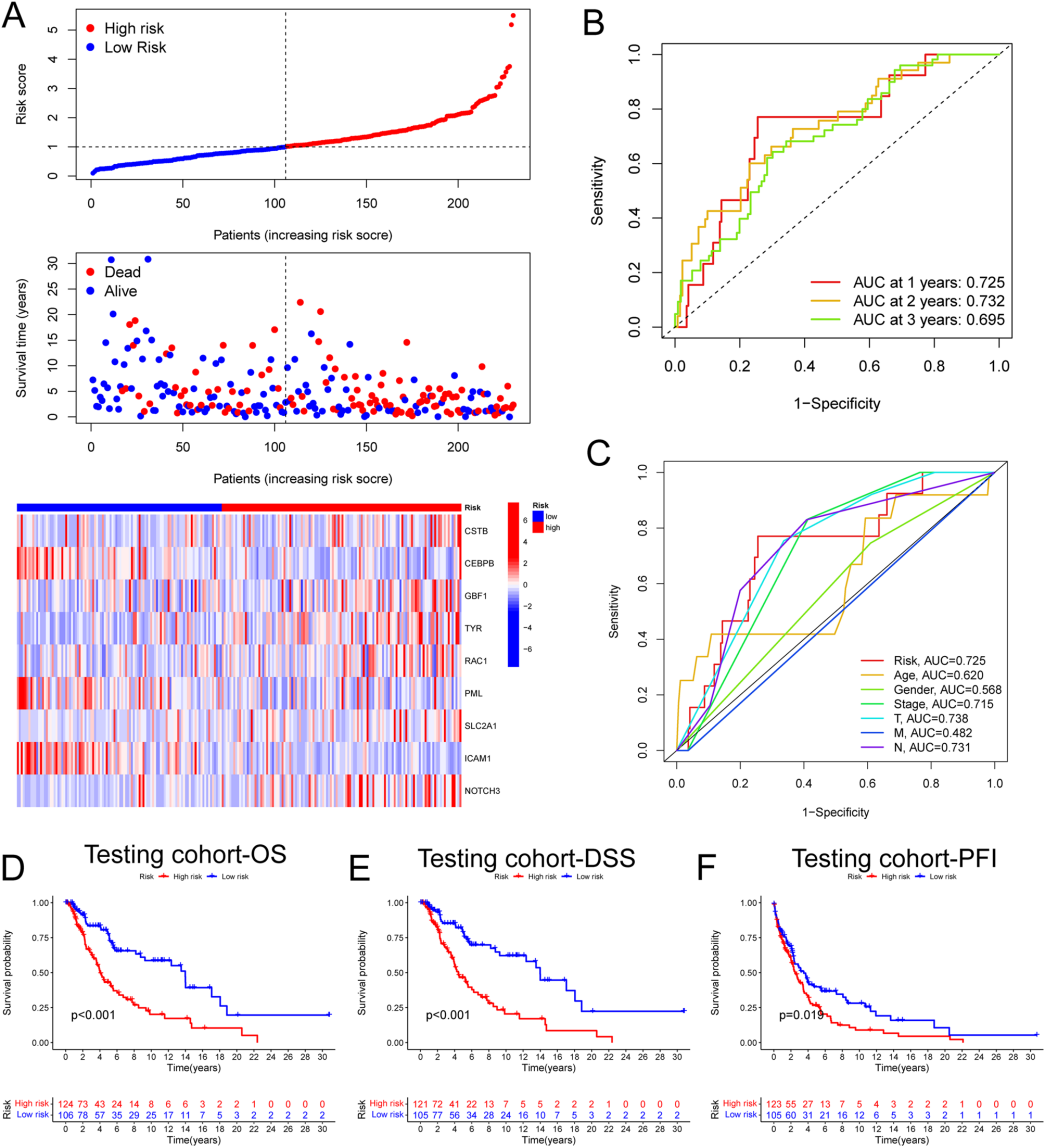
The prognostic value of the risk score in the internal testing set. (**A**) The risk curve and scatterplot based on the risk score and survival status of each melanoma sample. Besides, the heatmap showed the expression levels of ERGs in the high-risk and low-risk groups. (**B**) The AUC for the risk score at 1-, 2- and 3 years according to the ROC curves. (**C**) The AUC for the risk score and other clinical features at 1-year according to the ROC curves. The Kaplan–Meier survival analysis showed the OS (**D**), the DSS (**E**), and the PFI (**F**) of melanoma patients between high- and low-risk groups. OS, overall survival. PFI, progression-free interval. DSS, disease-specific survival.

In the training set, based on the median threshold of risk score, a total of 458 melanoma patients were divided into low risk group (229 patients) and high risk group (229 patients) (Fig. 2A). In the testing set, a total of 230 melanoma patients were divided into low risk group (106 patients) and high risk group (124 patients) in the same way (Fig. 3A). The risk score distribution of the sample demonstrated that ﻿the proportion of deaths of samples with high risk scores was significantly higher than that with low risk scores both in the training and testing set, which validated that high risk score predicted wore prognosis of melanoma patients (Figs. 2A, 3A). The time-dependent ROC curves were drawn to validate the reliability and prognostic value of the nine-ERG signature. In the training set, the AUC values of 1, 2 and 3 years were 0.771, 0.747 and 0.702 respectively (Fig. 2B). In the testing set, the AUC values of 1, 2 and 3 years were 0.725, 0.732 and 0.695 respectively (Fig. 3B). These results indicated the good predictive ability of the risk score in melanoma patients. Furthermore, in the training set, the AUC value of the risk score at 1-year was higher than other clinical factors, including gender, age, clinical stage and TNM stages, which also revealed the optimal predictive ability of the risk score in melanoma prognosis (Fig. 2C). We could find the similar tendency in the internal and external testing set (Figs. 3C, S1). Importantly, both in the training and testing set, the Kaplan–Meier survival analysis unraveled that the OS (Figs. 2D, 3D), disease-specific survival (DSS) (Figs. 2E, 3E) and progression-free interval (PFI) (Figs. 2F, 3F) of the melanoma patients in the high risk group was significantly shorter than those patients in the low risk group (P < 0.05).

### Independent prognostic value of the risk score

**Figure 4:**
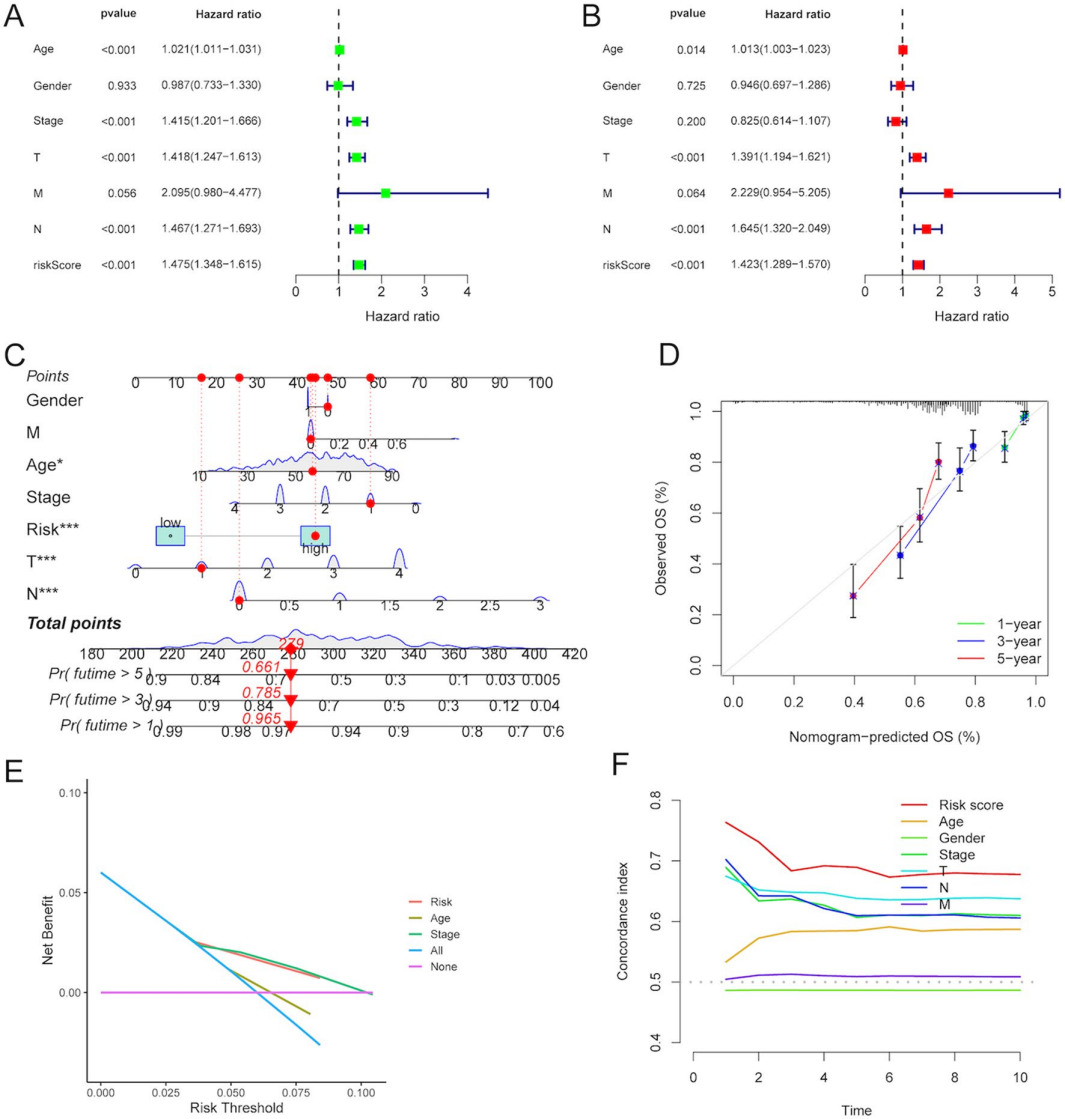
The independent predictive ability of the risk score. The univariate (**A**) and multivariate (**B**) Cox regression analysis of the risk score and other clinical features regarding prognostic value. (**C**) Development of a nomogram enrolling risk score and other clinical features to predict 1-, 3-, and 5-year OS of melanoma patients. (**D**) The calibration plots of the nomograms based on the agreement between nomogram-predicted and observed 1-, 3- and 5-year survival outcomes of melanoma. (**E**) DCA of the risk score and other clinical variables for predicting 1-year OS of melanoma patients. (**F**) Time-dependent C-index value of the risk score and other clinical features. C-index, concordance index. ﻿DCA, decision curve analysis.

The risk score together with all available clinical variables, including age, gender, clinical stage and TNM stages, were enrolled to execute univariate and multivariate Cox regression analyses. The result of univariate Cox regression analysis showed that the risk score was an independent factor with prognostic value of melanoma patients (HR = 1.475, 95% CI = 1.348–1.615, P < 0.001) (Fig. 4A). Also, in the multivariate Cox regression analysis, the risk score could serve as a good predictor of independence in melanoma prognosis (HR = 1.423, 95% CI = 1.289–1.570, P < 0.001) (Fig. 4B). Next, we recruited all independent predictors to construct a nomogram (Fig. 4C). Fortunately, the calibration plot for the prediction of 1-, 3- and 5-year OS was ideally consistent with actual observation (Fig. 4D). Decision curve analysis (DCA) unraveled that compared to other clinical variables, the risk score and clinical stage were superior in predicting 1-year OS of melanoma patients (Fig. 4E). Of note, the time-dependent C-index of the risk score was higher than other clinical variables, which demonstrated that the prediction accuracy of the prognostic risk score was greater than age, gender, clinical stage and TNM stages (Fig. 4F).

### Comparison of the risk signature with other external signatures

**Figure 5:**
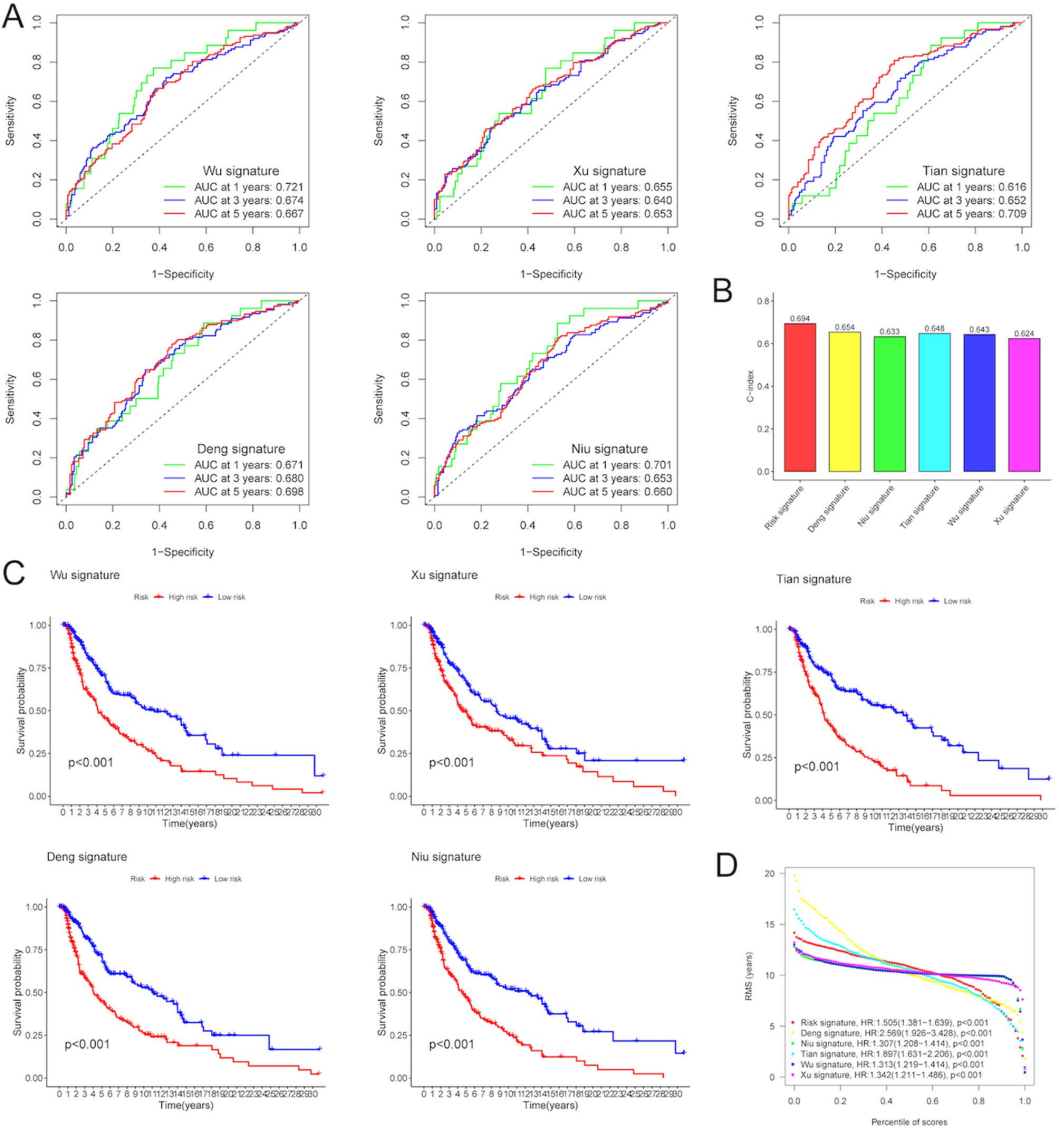
Comparison of the risk signature with other external signatures. (**A**) The ROC curves of Wu’s signature, Xu’s signature, Tian’s signature, Deng’s signature and Niu’s signature. (**B**) C-index of the 6 prognostic risk signatures. (**C**) The Kaplan–Meier survival curves of melanoma patients in high- and low-risk groups based on 5 external risk scores. (**D**) The HR of the risk score in 6 signatures. C-index, concordance index. HR, hazard ratio.

By reviewing literatures, five external prognostic signatures were selected to verify the reliability and stability of the ERGs-based signature, including Wu’s signature^15^, Xu’s signature^16^, Tian’s signature^17^, Deng’s signature^18^ and Niu’s signature^19^. All those external risk signatures were used to predict melanoma prognosis, and to make the comparison feasible, the process of calculating the risk score stayed the same as ours. As shown in Fig. 5A, the AUC value of all these five external risk signatures at 1-year was lower than our ERGs-based signature. Besides, the C-index of our risk signature was the highest, confirming the optimal prediction performance of the risk score (Fig. 5B). The Kaplan–Meier analysis showed that in those five external signatures, the OS of melanoma patients in the high-risk group was significantly shorter than those in the low-risk group (P < 0.001) (Fig. 5C). In addition, all the HR of the risk score was > 1, indicating that in these 6 signatures, the risk score was a risk factor for melanoma prognosis (Fig. 5D).

### Correlation of the risk score with melanoma clinical characteristics

**Figure 6:**
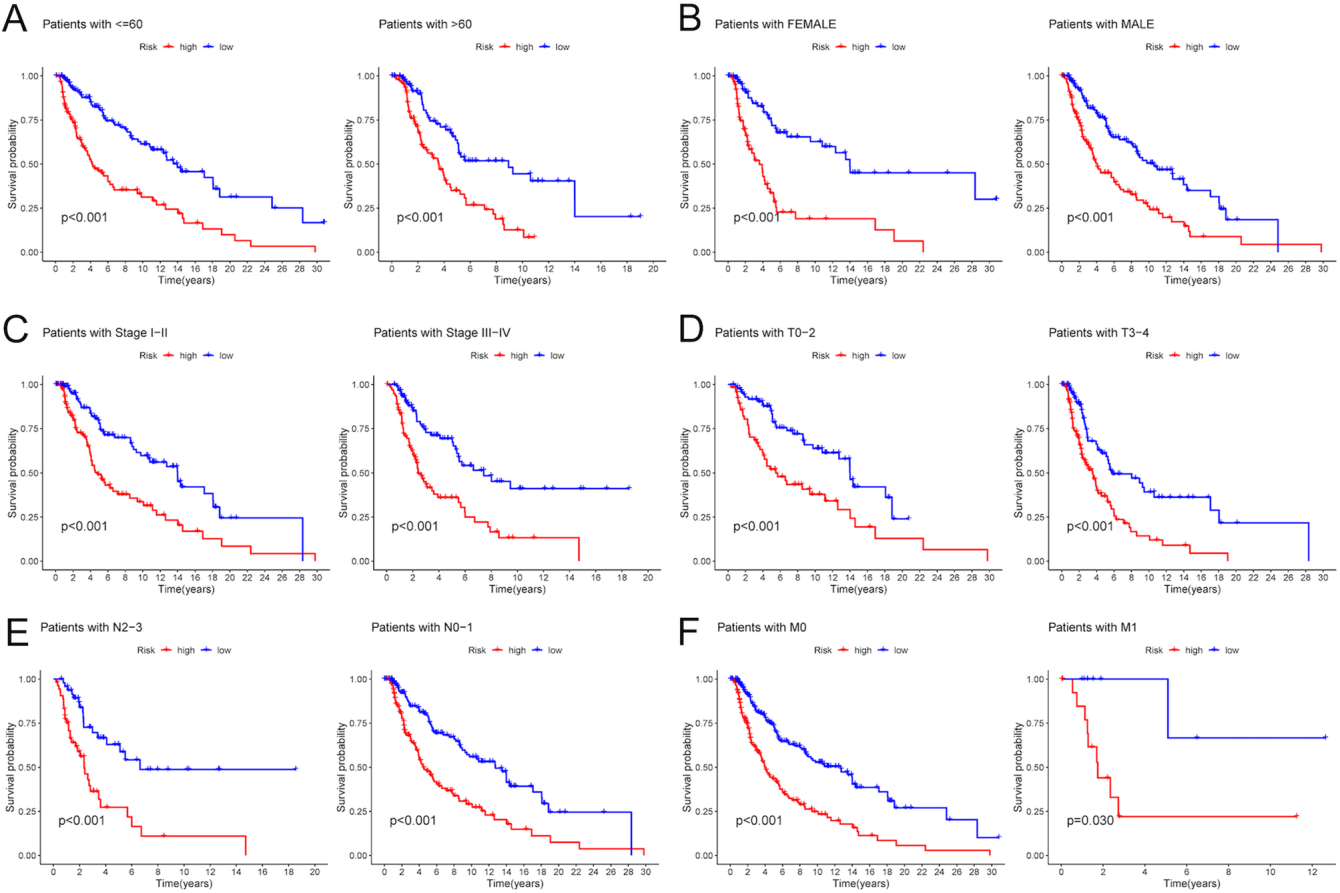
Correlation of the risk score with clinical characteristics. The Kaplan–Meier survival curves of melanoma patients with age > 60 or age ≤ 60 (**A**), female or male (**B**), stage I-II or stage III–IV (**C**), T0-2 or T3-4 (**D**), N0-1 or N2-3 (**E**), and M0 or M1 (**F**).

To explore the clinical appliance of our risk score, the survival time of melanoma patients with different clinical features in high- and low-risk groups were compared by using the Kaplan–Meier analysis. The survival probability of melanoma patients in the high-risk group was significantly lower than those in the low-risk group with age > 60, age ≤ 60, female, male, stage I–II, stage III–IV, T0–T2, T3–T4, N0–N1, N2–N3, M0 and M1 (P < 0.05) (Fig. 6A–F). These results unraveled the strong correlation of our risk score with clinical signs.

### Differential immune conditions between two risk subgroups

**Figure 7:**
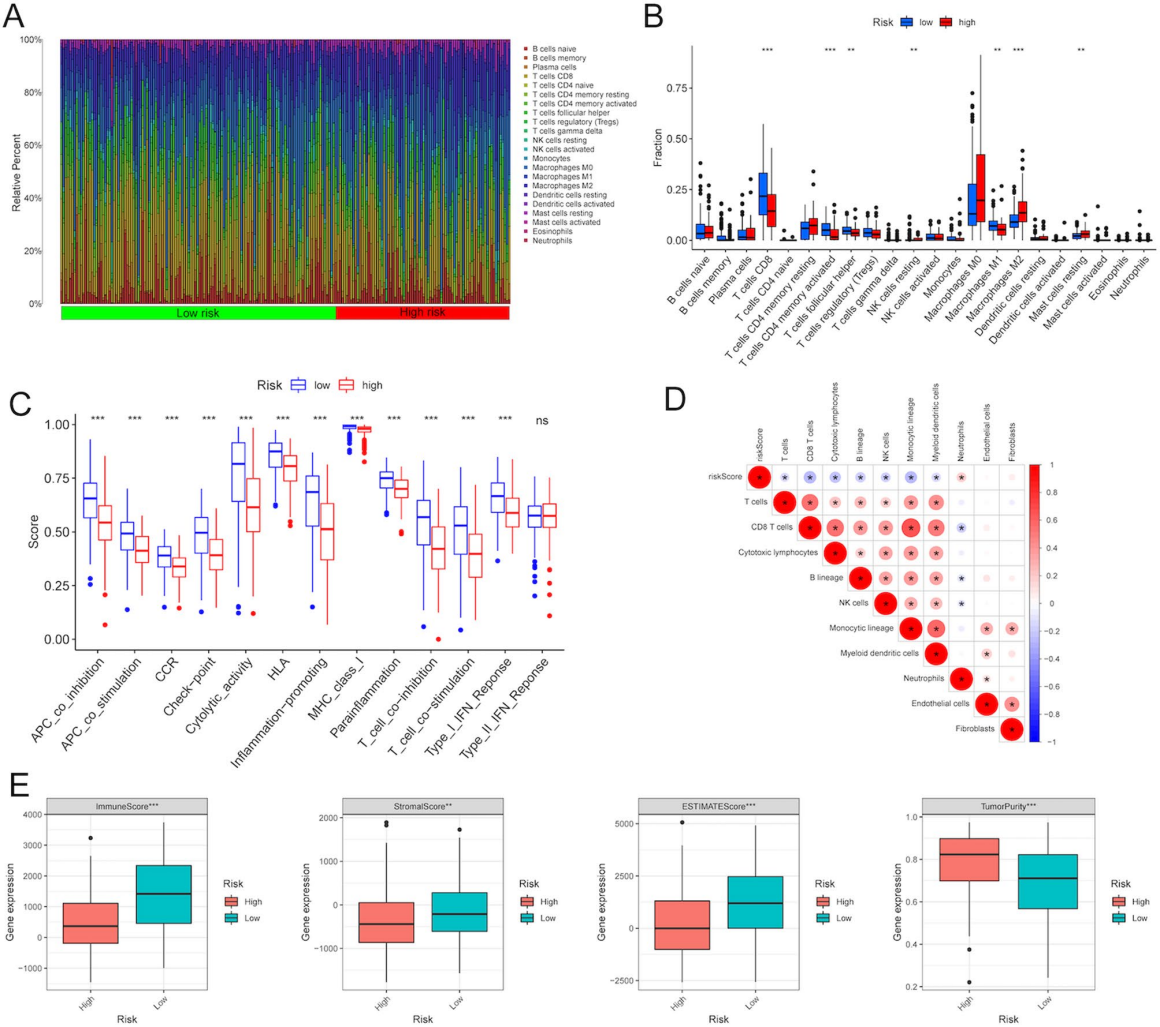
Differential immune state of patients between high- and low-risk groups. (**A**) Relative proportions of infiltration for 22 immune cells in the high- and low-risk groups. (**B**) Difference of infiltrating immune cell subtypes and expression levels between the high- and low-risk groups. (**C**) The distinction of enrichment of immune-related function between the high- and low-risk groups. (**D**) The Pearson correlation analysis about the relationship between risk score and immune cells. (**E**) Comparison of the ESTIMATE algorithm (estimate score, stromal score and immune score) and tumor purity between the high- and low-risk groups. *P < 0.05, **P < 0.01; ***P < 0.001.

As the immune microenvironment plays crucial roles in the development of melanoma, we then investigated the differences in tumor immunity between two risk subgroups. ﻿The proportions of 22 types of infiltrating immune cells between two risk groups were illustrated in Fig. 7A. Meanwhile, by utilizing the CIBERSORT algorithm, the box plot was drawn to analyze the distribution of 22 types of immune cells in Fig. 7B. The low-risk group was mainly enriched in CD8+ T cells, activated memory CD4+ T cells, follicular helper T cells and M1 macrophages, while the high-risk group was mainly enriched in M2 macrophages. Moreover, by using the ssGSEA to quantify the enrichment scores of 13 immune cell-related functions, we found that the scores of the immune functions, such as APC co-inhibition, CCR, cytolytic activity, check-point, and inflammation-promoting, were significantly activated in the low-risk group (Fig. 7C). In addition, the risk score was significantly correlated with immune cells (Fig. 7D). Subsequently, given the complexity and diversity of the tumor microenvironment, we further explored the immune score, ﻿the stromal score, the tumor purity, and the ESTIMATE score (stromal score combined with immune score) between two risk groups. As shown in Fig. 7E, the low-risk group had higher immune score, stromal score and ESTIMATE score, but had lower tumor purity. These results revealed that patients with different risk scores displayed a significantly different immune characterization. Patients with low-risk scores were prone to immune-excluded conditions accompanied by stromal activation and abundant immune infiltration, while patients with high-risk scores were characterized by a decreased immune infiltration.

### Differential response to ICI treatment of melanoma patients in two risk groups

**Figure 8:**
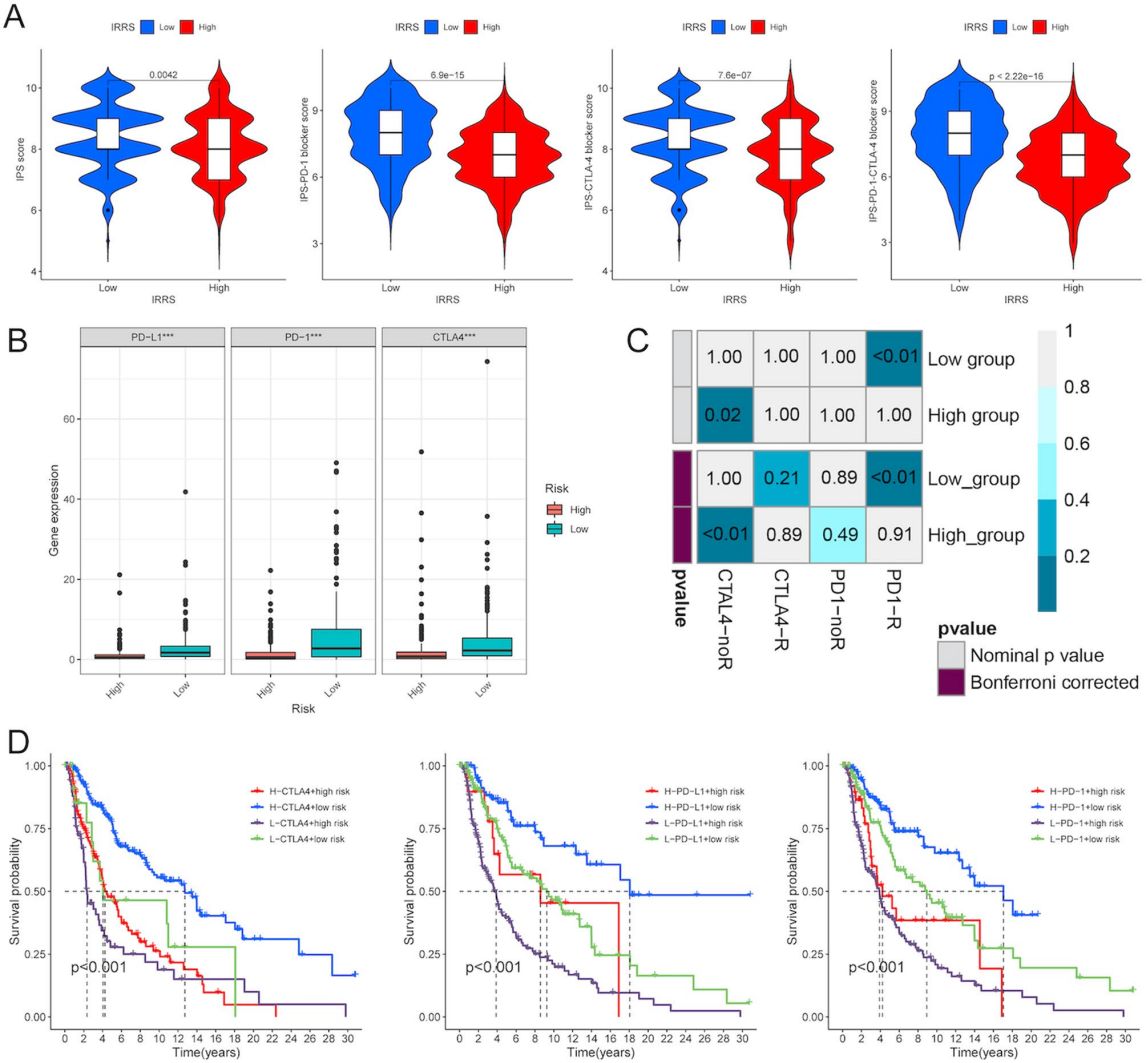
The response of patients with high- and low-risk scores to ICI treatment. (**A**) The distribution plot of IPS score, IPS-PD-1 blocker score, IPS-CTLA-4 blocker score and IPS-PD-1-CTLA-4 blocker score. (**B**) The expression levels of PD-1, PD-L1 and CTLA4 between the high- and low-risk groups. (**C**) SubMAP analysis of the possibility of anti-PD1 and anti-CTLA4 response between the high- and low-risk groups. (**D**) The Kaplan–Meier curves of melanoma patients with the combination of different risk scores with different expression levels of PD-1/PD-L1 or CTLA4. *P < 0.05, **P < 0.01; ***P < 0.001. ICI, immune checkpoint inhibitors. IPS, immunophenoscore. PD-1, programmed death-1. PD-L1, programmed death-ligand 1. CTLA4, cytotoxic T-lymphocyte-associated protein 4.

Antibodies against ICIs including the CTLA4 receptor and PD-1 receptor have dramatically revolutionized the clinical outcomes for melanoma patients with poor prognosis^20^. So, four types of IPS values, including PD-1-positive score or CTLA4-positive, were used to explore the prediction for the immunotherapeutic outcome of melanoma patients in high- and low-risk groups. As expected, IPS score, IPS-PD-1 blocker score, IPS-CTLA4 blocker score and IPS-PD-1-CTLA4 blocker score were higher in melanoma patients with low risk scores (all P < 0.001) (Fig. 8A), elucidating that melanoma patients in the low-risk group might benefit more from immunotherapy. Furthermore, the expression of PD-1, PD-L1 and CTLA-4 were higher in patients in the low-risk group (Fig. 8B). Then, we used the SubMAP algorithm to investigate the sensitivity to anti-PD1 and anti-CTLA4 immunotherapy in the high- and low-risk groups. The result indicated that patients with low risk scores showed promising responses to anti-PD-1 therapy, while patients with high risk scores showed no significant response to anti-CTLA4 therapy (P < 0.01) (Fig. 8C). Notably, we next explore the difference in the survival time of melanoma patients by a combination of risk score with PD-1/PD-L1-CTLA-4. The Kaplan–Meier analysis showed that melanoma patients with the high expression of PD-1/PD-L1-CTLA4 and low-risk scores had the longest survival time (Fig. 8D). These results manifested that the risk score could serve as an optimal predictor in immunotherapy, and patients with low-risk scores exhibited a better response to immune treatment.

### Differential response to immunotherapy and differential sensitivity to chemo-drugs of melanoma patients with high- and low- risk scores

**Figure 9:**
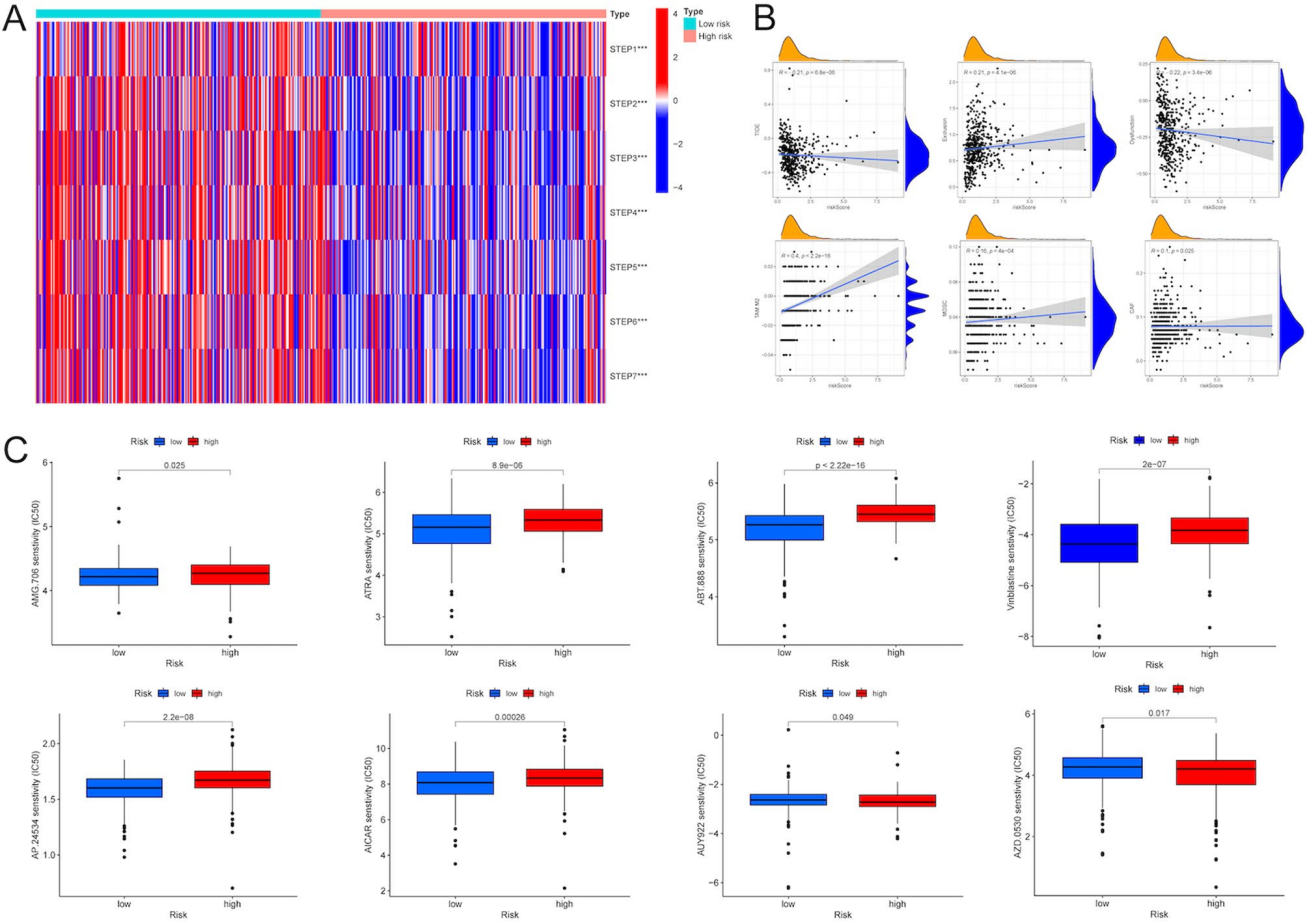
The estimation of the risk score in immunotherapy response and chemotherapy sensitivity. (**A**) The heatmap visualized the scores of seven steps of the cancer-immunity cycle between the high- and low-risk groups. (**B**) Correlation analysis between risk score and TIDE scores, including CAF, MDSCs, TAM, dysfunction of CTL, and exclusion of CTL. (**C**) The IC50 value of commonly used chemotherapeutic drugs including AMG.706, ATRA, ABT.888, vinblastine, AP.24534, AICAR, AUY922 and AZD.0530 between the high- and low-risk groups. *P < 0.05, **P < 0.01; ***P < 0.001. TIDE, tumor immune dysfunction and exclusion. CAF, cancer-associated fibroblast. MDSCs, myeloid-derived suppressor cells. TAM, tumor-associated macrophage. CTL, cytotoxic T lymphocyte. IC50, 50% inhibitory concentration.

The effective outcome of the anticancer immune response was to kill cancer cells, which needs a series of stepwise events to proceed iteratively, namely the cancer-immunity cycle^21^. Firstly, cancer cell antigens are released caused by the death of cancer cells and captured by dendritic cells (DCs) (Step 1). Some specific immune signals, such as proinflammatory cytokines, are activated to induce peripheral tolerance to the tumor antigens. Then, DCs present the captured antigens to T cells (Step 2), and the T cells are stimulated by the recognition of the complex on the surface of the APC (Step 3). The activated T cells migrate (Step 4) and infiltrate (Step 5) the tumor bed. Next, through the interaction between T cell receptor (TCR) and cognate antigen, T cells recognize cancer cells specifically (Step 6) and kill the targeted cancer cells (Step 7). Hence, the death of the cancer cells releases the neoantigens to trigger the tumor-immunity cycle again. The score of each step was quantified by calculating the ssGSEA score of vital regulatory genes of each step. As shown in Fig. 9A, the seven steps in the low-risk group had higher scores than the high-risk group, which proposed that in the patients with low-risk scores, the cancer-immunity cycle might sustain an optimal initiation or reinitiation conditions helpful to cancer immunotherapy. Moreover, the TIDE algorithm was used to evaluate the universal applicability of the risk score to predict the responsiveness to immunotherapy (Fig. 9B). The result showed that the risk score was negatively correlated with dysfunction (r = − 0.22, P = 3.4e−06), while positively correlated with exclusion (r = 0.21, P = 4.1e−06). Besides, the risk score was positively correlated with the levels of tumor-associated macrophage (TAM) M2 (r = 0.4, P < 2.2e−16), myeloid-derived suppressor cell (MDSC) (r = 0.16, P = 4e−04), and cancer-associated fibroblast (CAF) (r = 0.1, P = 0.025). Particularly, the risk score was significantly correlated with TIDE (r = − 0.21, P = 6.8e−06). That was, patients with lower risk scores got higher TIDE scores, which could benefit more from immunotherapy. This conclusion was consistent with the result of the cancer-immunity cycle.

Chemotherapy has been a universal way for melanoma treatment. Hence, we next assessed the response to various drugs of melanoma patients in high- and low-risk groups. As shown in Fig. 9C, patients with low-risk scores got lower IC50 score for the majority of chemo-drugs, including AMG.706, ATRA, ABT.888, vinblastine, AP.24534, AICAR (P < 0.01), manifesting that patients in the low-risk group were more sensitive to chemotherapeutics.

### Validation of the expression profiles of the nine risk ERGs

**Figure 10:**
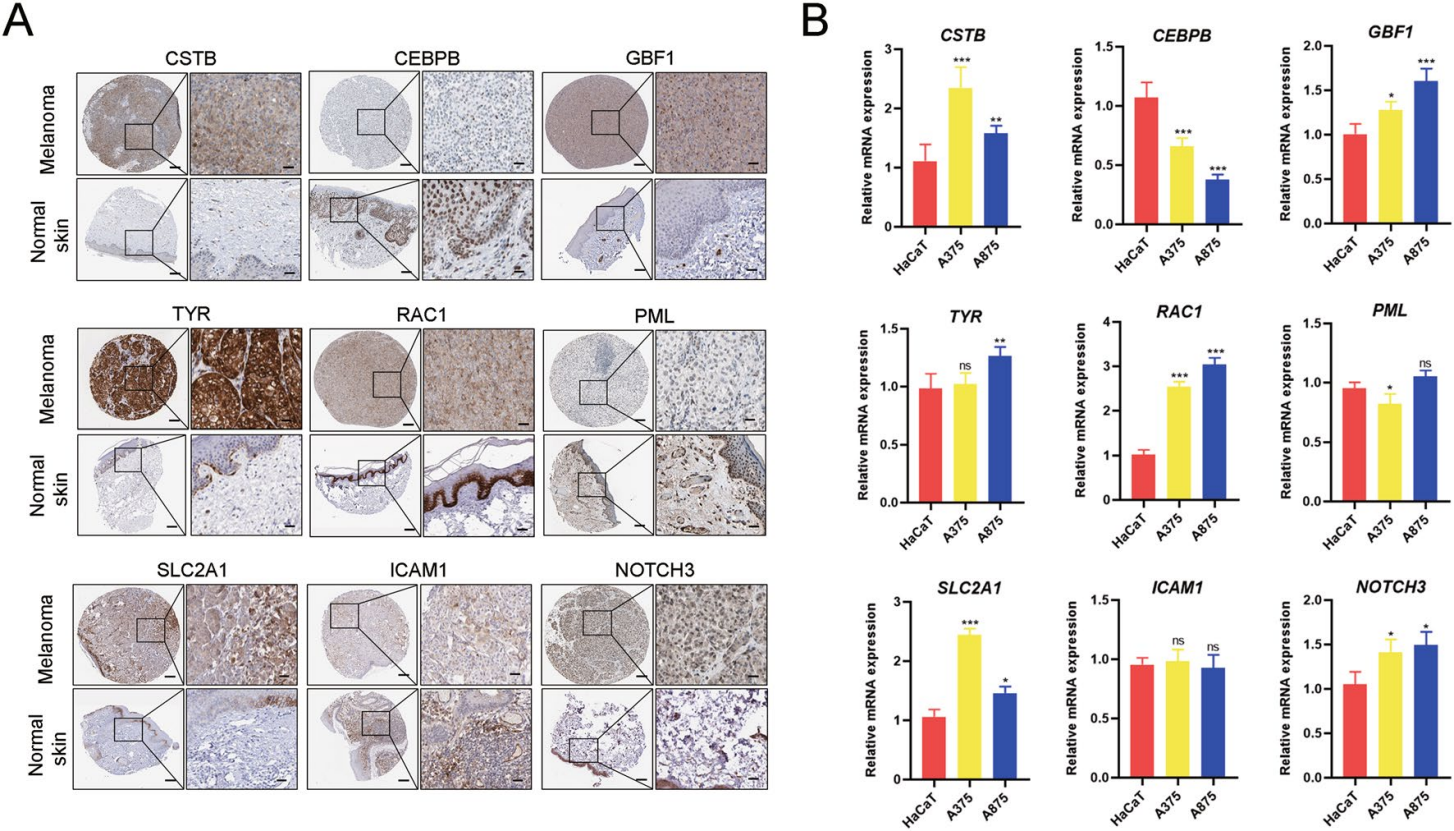
Verification of the differential expression profiles of the nine ERGs in melanoma samples. (**A**) The immunohistochemical staining images of nine ERGs-coding proteins. (**B**) The qPCR results showed the differential expressions of nine ERGs in HaCaT, A375 and A875 cells. *P < 0.05, **P < 0.01; ***P < 0.001. ns, no significance.

The expression profiles of nine ERGs in the signature were further analyzed. The IHC staining images for these genes coding proteins were obtained from the HPA database. The results revealed that the CSTB, GBF1, TYR, RAC1, SLC2A1 and NOTCH3-coding proteins were significantly enriched in melanoma samples, while CEBPB-coding protein had low expression in melanoma samples (Fig. 10A). Furthermore, the expression profiles of PML and ICAM1 coding proteins show no significance. Next, qPCR was used to detect these gene expressions at the mRNA level in HaCaT cells (negative control) and melanoma cells. Consistently, the nine ERG-expression profiles were consistent with the corresponding coding proteins in melanoma samples (Fig. 10B). Notably, these findings brought into correspondence with the differential expression landscapes of nine ERGs between normal and tumor samples, thus verifying the reliability of the risk ERG signature.

### Inhibition of RAC1 suppressed the proliferation and migration and promoted the apoptosis of melanoma cells as well as up-regulated PD-1/PD-L1 and CTLA4 levels in melanoma cells

**Figure 11:**
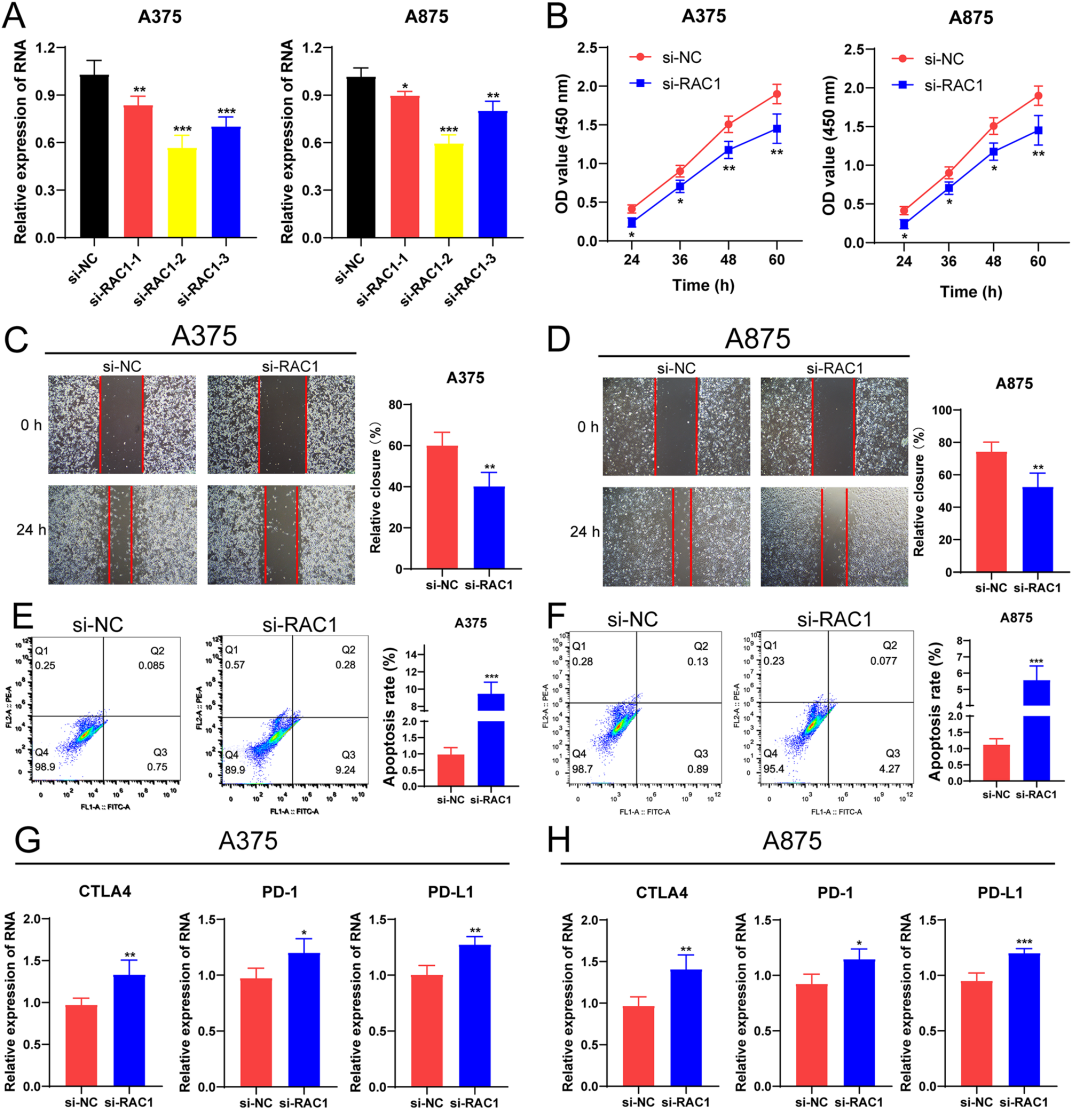
Inhibition of RAC1 affected melanoma cell behavior and expression levels of PD-1/PD-L1 and CTLA4. (**A**) qRT-PCR analysis of RAC1 expression in A375 and A875 cells. (**B**) The CCK8 assay about the proliferation of A375 and A875 cells after transfection with si-NC or si-RAC1. The scratch/wound healing assay about the migrations of A375 cells (**C**) and A875 cells (**D**) after transfection with si-NC or si-RAC1 for 24 h. The flow cytometry assay about the apoptosis rate of A375 cells (**E**) and A875 cells (**F**) after transfection with si-NC or si-RAC1. Si-NC, siRNA negative control. qRT-PCR analysis of PD-1/PD-L1 and CTLA4 expression in A375 cells (**G**) and A875 cells (**H**). *P < 0.05, **P < 0.01; ***P < 0.001 vs. siRNA negative control.

In our study, nine ERGs were successfully used to calculate the risk score, including CSTB, CEBPB, GBF1, TYR, RAC1, PML, SLC2A1, ICAM1 and NOTCH3. Among them, the high expression of RAC1 was associated with a shorter survival time of melanoma patients, and whether RAC1 could influence the behavior of melanoma cells has not been studied before. Therefore, we intended to explore the functional role of RAC1 in melanoma cells, A375 and A875 cells. Firstly, ﻿the RNA expression of RAC1 could be effectively decreased by siRNA in A375 and A875 cells (Fig. 11A). The result from the CCK-8 assay showed that the proliferation of A375 and A875 cells was significantly suppressed by the inhibition of RAC1 (Fig. 11B). Then, the scratch/wound-healing assay indicated that the restraint of RAC1 could attenuate the migration of A375 cells and A875 cells (Fig. 11C, D). Furthermore, flow cytometry analysis elucidated that the apoptotic rates of A375 cells and A875 cells were dramatically upregulated in RAC1 knockdown cells (Fig. 11E, F). These results showed that RAC1 could act as an accelerator in melanoma progression by promoting the proliferation and invasion and repressing the apoptosis of melanoma cells. As we confirmed that the patients in the low-risk group with higher expression of PD-1/PD-L1 and CTLA4, had a better response to immunotherapy, we intended to excavate whether the RAC1 could affect the therapeutic efficiency by influencing the expression of PD-1/PD-L1 and CTLA4. As expected, the RNA levels of CTLA1, PD-1/PD-L1 were significantly augmented in RAC1 knockdown melanoma cells by qRT-PCR analyses (Fig. 11G, H).

## Discussion

The prognosis and survival of melanoma patients, the most aggressive type of skin cancer, still remain a challenge. Moreover, patients’ responses to chemotherapy and immunotherapy are still poor^22^. Therefore, the pursuit of molecular biomarkers and therapeutic targets with prognostic and predictive value might bring out promising improvement for OS and cure rates of patients with melanoma. Many prognostic signatures have been constructed to predict the prognosis of melanoma patients. For example, Huang et al.^23^ found immune-related biomarkers associated with melanoma prognosis and tumor microenvironment. In the study of Tian et al.^24^, a seven-gene signature composed of immune checkpoints was identified to predict prognosis and immunotherapy in melanoma patients. In 2021, Liu et al.^25^ established a six-gene signature related to glycolysis and immune response in uveal melanoma. Whereas, the predictive performance of these biomarkers is limited and more accurate and novel signatures are needed to excavate (Fig. 11).

Malignant tumor cells could thrive even under adverse conditions, including dysregulated proliferation, oxidative stress, nutrient and lipid deprivation, hypoxia, and acidic extracellular pH^26^. The unfavorable microenvironmental conditions could hinder the protein-folding process in the ER, thereby provoking a cellular state of ER stress^27^. Importantly, multiple pro-tumoral attributes could be governed by a state of persistent ER stress^28^. Meanwhile, the aberrant stimulation of ER stress has been considered as a key modulator in tumor progression and sensitivity to immunotherapy and chemotherapy. However, the predictive ability of the ERGs in melanoma prognosis needs further exploration.

In the present study, a novel nine-ERG signature, including CSTB, CEBPB, GBF1, TYR, RAC1, PML, SLC2A1, ICAM1 and NOTCH3, was built to effectively predict the prognosis of patients with melanoma. Five of nine identified ER stress-related genes, CEBPB, TYR, SLC2A1, NOTCH3 and RAC1, have been reported to engage in melanoma malignant behavior regulation. CEBPB, a member of the CCAAT/enhancer binding protein (CEBP) family of transcription factors, mediates cellular events including energy metabolism, cell proliferation and differentiation, and regulates inflammatory stimulation and immunity functions^29,30^. The augment of CEBPB significantly increased melanoma cell apoptosis and death, especially for melanoma cells resistant to the BRAFi vemurafenib^31^. Tyrosinase (TYR), a copper-containing monooxygenase, catalyzes various phenols into melanin pigments and other polyphenolic compounds^32^. The increased expression of TYR might serve as a salient marker of the low potential for metastasis in melanoma, owing to the downregulation of melanoma cell migration, cell survival, epithelial-mesenchymal transition, and tumorigenesis^33^. The glucose transporter isoform 1 (GLUT1; SLC2A1) has been reported to promote aggressive tumor growth for functioning as a key rate-limiting factor in transporting glucose into cancer cells^34^. Consistently, GLUT1 suppression significantly reduced melanoma cell proliferation, apoptosis resistance and migratory activity, unraveling the favorable role of GLUT1 in melanoma cell metastatic behavior^35^. NOTCH3 belongs to the classic Notch family and acts as signaling receptors to dominate cell fate in many tissue contexts^36^. NOTCH3 mediates the communication between melanoma cells and endothelial cells, thus promoting tumor cell migration^37^. Induced NOTCH3 signaling in melanoma cells enhanced tumor cell migration while causing no significant increases in tumor cell growth. Moreover, NOTCH3 was considered as a molecular switch driving melanoma heterogeneity since the knockdown of NOTCH3 in melanoma cell lines retarded and abolished tumorigenicity. However, the roles of CSTB, GBF1, PML and ICAM1 in melanoma development were still unclear, which deserved to further exploration. Our study constructed a predictive risk signature based on nine ERGs. The OS, DSS and PFI of melanoma patients with high-risk scores were significantly shorter than those with low-risk scores. And, there existed significant distinction of survival time of patients with clinical characteristics in high- and low-risk groups. Meanwhile, our risk score could serve as a predictor of independence in melanoma and the nomogram including the risk score performed well in predicting 1-, 3-, and 5-year OS of melanoma patients. The C-index of our nine-gene signature was the highest compared to other risk signatures. These findings supported that the risk signature based on ERGs played a vital role in predicting melanoma prognosis.

The tumor microenvironment (TME), a complex network of stromal cells comprised of fibroblasts, vascular cells and inflammatory immune cells, plays crucial roles in promoting cancer growth and invasion^38^. Immunity plays anti-tumorigenic function through immunosurveillance and immunological sculpting of tumor heterogeneity. Alternatively, by exerting direct tumor-promoting signals on cancer cells, pro-tumorigenic inflammation creat a tumor-permissive state and favors cancer progression^39^. In our study, patients with low-risk scores had more abundant infiltration of immune cells and more activation of immune functions than those with high-risk scores. Meanwhile, patients in the low-risk group got higher immune score, stromal score, ESTIMATE score and lower tumor purity. That is, the low-risk score tended to induce an activated immune state in the TME and stimulate the death of cancer cells for melanoma patients. Besides, the management of cancer patients was a challenge by advances in immunological treatment, and immune checkpoint molecules, including the PD-1 and CTLA4, govern immune responses within the TME^40^. Notably, the appliance of novel treatment strategies, mainly including ICIs, has substantially improved the prognosis of patients with metastatic melanoma^41^. In our models, the low-risk-group patients with melanoma enriched higher expression of PD-1/PD-L1 and CTLA4, and showed promising responses to anti-PD-1 therapy. Additionally, patients with low-risk scores got higher IPS value of PD-1-positive or CTLA4-positive. Furthermore, melanoma patients with the high expression of PD-1/PD-L1-CTLA-4 and low-risk scores had the longest survival time. These results unravel that the risk score could serve as an additional tool for adjuvant ICI treatment in melanoma. In addition, patients with low-risk scores got higher scores of cancer-immunity cycle and higher TIDE score, which further validated that low-risk-group patients displayed more stimulated antitumor immune responses and could benefit more from immunotherapy.

Among those nine ERGs, patients with high expression of RAC1 had shorter survival time. RAC1 is a small GTPase that functions in modulating cell shape, motility, survival, and division^42^. The RAC1 protein has long been recognized as a central signaling hub that participates in transformation by many oncogenes. In melanoma, selective PI3Kβ inhibitors could inhibit the growth and migration of melanoma cell lines induced by mutant RAC1^43^. Colón-Bolea et al.^44^ found that RAC1 enhanced melanoma invasiveness by inducing nuclear alterations through the LINC complex. In our study, we validated that the suppression of the RAC1 expression indeed attenuated the proliferation and migration as well as augmented the apoptosis of melanoma cells, which was consistent with the conclusions reported in other studies. Alternatively, the expression of the immune checkpoint, PD-1/PD-L1 and CTLA4, was augmented in the melanoma cells with the knockdown of RAC1. This meaningful and interesting discovery of RAC1 influences in immune checkpoints might provide additional therapeutic targets for immune treatment in melanoma.

There still exist some limitations of our study. The ethnicity, lifestyle, genetic background and disease subtypes influenced the incidence, mortality, prognosis and therapy response of melanoma patients^45^. It was more practical to analyze the ability of the risk signature in predicting the prognosis and immunotherapy response of melanoma patients with different subtypes. Besides, the predictive effect of the nine-ERG-signtaure was mainly confirmed from the online databases and lack of external validation in animal models or clinical cohorts. And, more complex mechanisms correlated with the prognosis of melanoma required further exploration. Meanwhile, the other key clinical factors that may affect the survival of melanoma patients have not been excluded. Still, our study only included the validation of RAC1 in melanoma cell proliferation, apoptosis and migration. It would be more reliable and meaningful to verify the functional role of the other 8 ERGs in the regulation of melanoma behavior.

## Conclusions

In summary, we successfully established a nine-ERG-signature for effectively predicting the prognosis of melanoma patients. Especially, the present study provided a potential association of ERGs with cancer immunity and immunotherapy. The risk signature constructed by ERGs can be used as an integrated predictor for melanoma prognosis and a ponderable reference for personalized immunotherapy.

## Supplementary Information

Supplementary Figure S1.

Supplementary Table S1.

Supplementary Table S2.

Supplementary Table S3.

Supplementary Table S4.

## Data Availability

All the datasets displayed in this study can be obtained in the online database. Further questions can be directed to the corresponding author.

## Abbreviations

C-index: Concordance index
ICI: Immune checkpoint inhibitor
IPS: Immunophenoscore
LASSO: Least Absolute Shrinkage and Selection Operator
MDSC: Myeloid-derived suppressor cell
PD-L1: Programmed death-ligand 1
ssGSEA: Single-sample gene set enrichment analysis
TCGA: The Cancer Genome Atlas
TAM: Tumor-associated macrophage
AUC: Area under the curve
BCA: Bicinchoninic acid
BSA: Bull serum albumin
CAF: Cancer-associated fibroblast
CCK-8: Cell counting kit-8
cDNA: Complementary DNA
CEBP: CCAAT/enhancer binding protein
CTLA4: Cytotoxic T-lymphocyte-associated antigen 4
DCA: Decision curve analysis
DCs: Dendritic cells
DSS: Disease-specific survival
DMEM: Dulbecco’s modified Eagle’s medium
ER: Endoplasmic reticulum
ECL: Enhanced chemiluminescence
ERGs: ER stress-related genes
GLUT1: Glucose transporter isoform 1
OD: Optical density
OS: Overall survival
PD-1: Programmed cell death-1
PFI: Progression-free interval
PI: Propidium iodide
qRT-PCR: Quantitative reverse transcription-polymerase reaction
RIPA: Radio-immuno-precipitation assay
ROC: Receiver operating characteristic
SDS-PAGE: Sodium dodecyl sulfate–polyacrylamide gel electrophoresis
SERP1: Stress-associated endoplasmic reticulum protein 1
TCR: T cell receptor
TBST: Tris-buffered saline containing Tween
TIDE: Tumor Immune Dysfunction and Exclusion
TME: Tumor microenvironment
TYR: Tyrosinase
IC50%: 50% Inhibitory concentration
