# Supplementary material for: A comprehensive signature based on endoplasmic reticulum stress-related genes in predicting prognosis and immunotherapy response in melanoma
Source: Sci Rep. 2023 May 22;13:8232. doi: 10.1038/s41598-023-35031-9 (PMC10203260; doi:10.1038/s41598-023-35031-9)
Supplement: Supplementary file 1 — Supplementary Figure S1. [file 41598_2023_35031_MOESM1_ESM.docx]

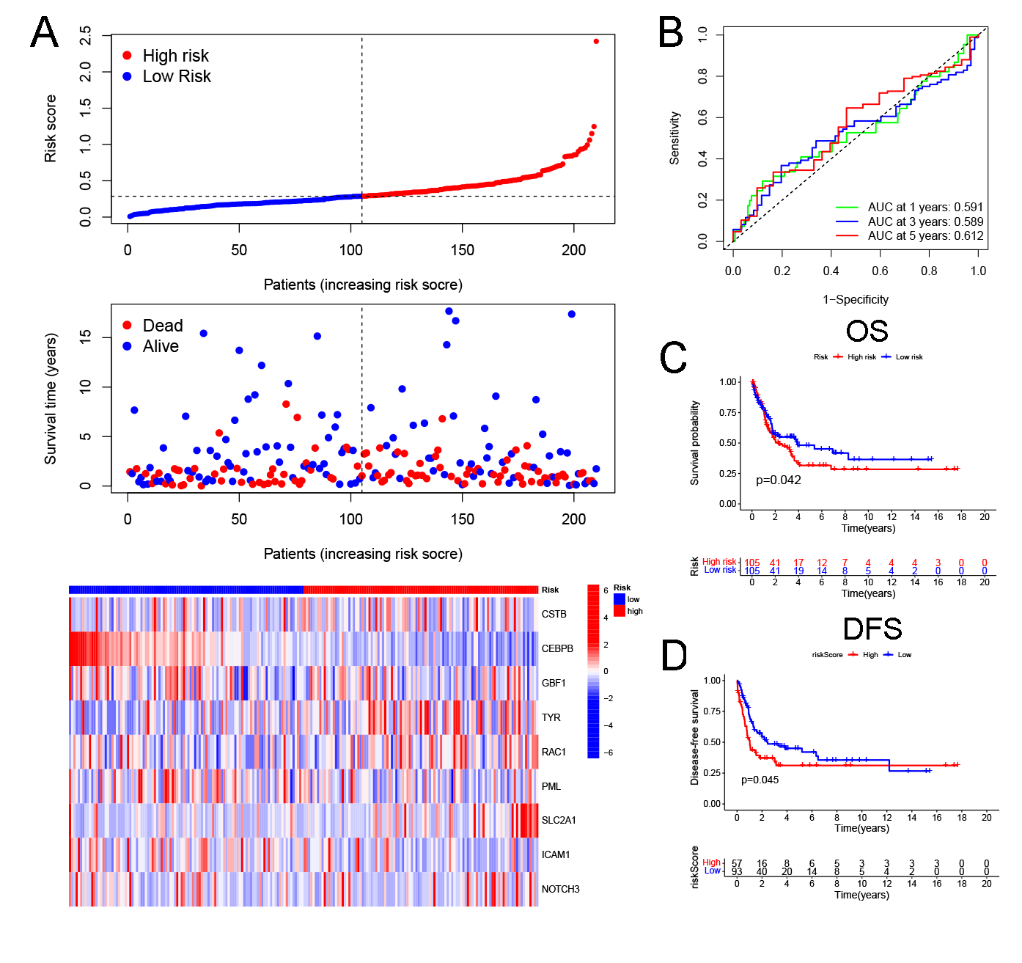


Figure S1. The prognostic value of the risk score in the external testing set. (A) ﻿The risk curve and scatterplot based on the risk score and survival status of each melanoma sample. Besides, the heatmap showed the expression levels of ERGs in the high-risk and low-risk groups. (B) The AUC for the risk score at 1-, 3- and 5 years according to the ROC curves. The Kaplan-Meier survival analysis showed the OS (C) and the DFS (D) of melanoma patients between high- and low-risk groups. OS, overall survival. DFS, disease-free survival.
