# Supplementary material for: A comprehensive signature based on endoplasmic reticulum stress-related genes in predicting prognosis and immunotherapy response in melanoma
Source: Sci Rep. 2023 May 22;13:8232. doi: 10.1038/s41598-023-35031-9 (PMC10203260; doi:10.1038/s41598-023-35031-9)
Supplement: Supplementary file 2 — Supplementary Table S1. [file 41598_2023_35031_MOESM2_ESM.docx]

**Table S1.** Summary of clinical characteristics of TCGA-SKCM dataset.

| **Characteristic** | **TCGA-SKCM data set (n = 458)** |
| --- | --- |
| **Vital status, n (%)** |  |
| Alive | 239 (52.1) |
| Dead | 219 (47.9) |
| **Age, n (%)** |  |
| < 65 | 287 (62.6) |
| ≥ 65  **Gender, n (%)**  Female  Male | 171 (37.4)  173 (37.8)  285 (62.2) |
| **WHO-Stage, n (%)** |  |
| 0  Ⅰ | 6 (1.3)  87 (19.0) |
| Ⅱ | 139 (30.3) |
| Ⅲ | 170 (37.1) |
| Ⅳ | 22 (4.8) |
| Unknow | 34 (7.5) |
| **AJCC-T stage, n (%)** |  |
| T0  T1 | 23 (5.0)  42 (9.2) |
| T2 | 76 (16.6) |
| T3 | 89 (19.4) |
| T4 | 152 (33.2) |
| TX  Unknow | 51 (11.1)  25 (5.5) |
| **AJCC-N stage, n (%)** |  |
| N0 | 229 (50.0) |
| N1 | 73 (15.9) |
| N2 | 49 (10.8) |
| N3 | 55 (12.0) |
| NX  Unknow | 35 (7.6)  17 (3.7) |
| **AJCC-M stage, n (%)** |  |
| M0 | 411 (89.7) |
| M1 | 23 (5.0) |
| MX | 24 (5.3) |
