# Supplementary material for: A comprehensive signature based on endoplasmic reticulum stress-related genes in predicting prognosis and immunotherapy response in melanoma
Source: Sci Rep. 2023 May 22;13:8232. doi: 10.1038/s41598-023-35031-9 (PMC10203260; doi:10.1038/s41598-023-35031-9)
Supplement: Supplementary file 3 — Supplementary Table S2. [file 41598_2023_35031_MOESM3_ESM.docx]

| Gene | Forward Primer | Reverse Primer |
| --- | --- | --- |
| RAC1 | ATGTCCGTGCAAAGTGGTATC | CTCGGATCGCTTCGTCAAACA |
| PD-1 | CCAGGATGGTTCTTAGACTCCC | TTTAGCACGAAGCTCTCCGAT |
| PD-L1 | TGGCATTTGCTGAACGCATTT | TGCAGCCAGGTCTAATTGTTTT |
| CTLA4 | GCCCTGCACTCTCCTGTTTTT | GGTTGCCGCACAGACTTCA |
| GAPDH | GGAGCGAGATCCCTCCAAAAT | GGCTGTTGTCATACTTCTCATGG |
| CSTB  CEBPB  GBF1  TYR  PML  SLC2A1  ICAM1  NOTCH3 | AGGTCCCAGCTTGAAGAGAAA  CTTCAGCCCGTACCTGGAG  TTGGGGCCATCAAACGAAATG  TGCACAGAGAGACGACTCTTG  CGCCCTGGATAACGTCTTTTT  GGCCAAGAGTGTGCTAAAGAA  ATGCCCAGACATCTGTGTCC  TGGCGACCTCACTTACGACT | CGCAGGTGTACGAAGTCCTC  GGAGAGGAAGTCGTGGTGC  TCAGAGCGAATCACTTCCAGA  GAGCTGATGGTATGCTTTGCTAA  CTCGCACTCAAAGCACCAGA  ACAGCGTTGATGCCAGACAG  GGGGTCTCTATGCCCAACAA  CACTGGCAGTTATAGGTGTTGAC |

**Table S2**. Primer sequences for genes.
